# Sulfiredoxin-1 blood mRNA expression levels negatively correlate with hippocampal atrophy and cognitive decline

**DOI:** 10.12688/f1000research.76191.2

**Published:** 2022-03-21

**Authors:** Catalina Anca Cucos, Ioana Cracana, Maria Dobre, Bogdan Ovidiu Popescu, Catalina Tudose, Luiza Spiru, Gina Manda, Gabriela Niculescu, Elena Milanesi

**Affiliations:** 1 Victor Babes National Institute of Pathology, Bucharest, 050096, Romania; 2 Medinst Diagnostic Romano-German SRL, Bucharest, Romania; 3 “Carol Davila” University of Medicine and Pharmacy, Bucharest, Romania; 4 Clinical Hospital Colentina, Bucharest, Romania; 5 Prof. Dr. Al. Obregia” Psychiatry Clinical Hospital & the Memory Center of the Romanian Alzheimer Society, Section II, Bucharest, Romania; 6 “Ana Aslan” International Foundation, Bucharest, Romania; 7 Faculty of Medical Engineering, University Politehnica of Bucharest, Bucharest, Romania

**Keywords:** SRXN1, MRI, blood, Cognitive decline

## Abstract

**Introduction:** Cognitive decline, correlating with hippocampal atrophy, characterizes several neurodegenerative disorders having a background of low-level chronic inflammation and oxidative stress.

**Methods:** In this cross-sectional study, we examined how cognitive decline and hippocampal subfields volume are associated with the expression of redox and inflammatory genes in peripheral blood. We analyzed 34 individuals with different cognitive scores according to Mini-Mental State Examination, corrected by age and education (adjMMSE). We identified a group presenting cognitive decline (CD) with adjMMSE<27 (n=14) and a normal cognition (NC) group with adjMMSE≥27 (n=20). A multiparametric approach, comprising structural magnetic resonance imaging measurement of different hippocampal segments and blood mRNA expression of redox and inflammatory genes was applied.

**Results:** Our findings indicate that hippocampal segment volumes correlate positively with adjMMSE and negatively with the blood transcript levels of 19 genes, mostly redox genes correlating especially with the left subiculum and presubiculum. A strong negative correlation between hippocampal subfields atrophy and Sulfiredoxin-1 ( *SRXN1*) redox gene was emphasized.

**Conclusions: **Concluding, these results suggest that *SRXN1* might be a valuable candidate blood biomarker for non-invasively monitoring the evolution of hippocampal atrophy in CD patients.

## 1. Introduction

Cognitive decline is a very early phase of several neurodegenerative disorders, including Alzheimer’s disease (AD) that is the main cause of dementia in the elderly population and affects 44 million people worldwide. ^1^

Brain degeneration can be evaluated using morphometric estimates obtained with magnetic resonance imaging (MRI). Hippocampus is particularly vulnerable to the aging processes, and its volume has been tied to decline in different cognitive areas including episodic, semantic, working memory, and visuospatial ability. ^2^ Indeed, hippocampal volume on a structural MRI scan represents one of the most valuable brain imaging markers used in clinical research to evaluate the severity and progression of AD. ^3^ In the last years, morphological changes within the hippocampus are being focused on the measurement of hippocampal subfields. A recent study conducted on subjects selected from Alzheimer’s Disease Neuroimaging Initiative (ADNI), investigating hippocampal subfield volumes, identified that atrophy of the bilateral CA1, CA2-CA4 and subiculum subfields was higher in AD patients compared to mild cognitive impairment (MCI) individuals and controls, and registered a high atrophy rate in whole hippocampus, CA1 and subiculum subfields of MCI. ^4^ Despite its high diagnostic potential, MRI displays discomforts and feasibility issues, occurring mostly in elderly individuals. ^5^^,^^6^

Molecular aberrations in the AD brain are reflected in the cerebrospinal fluid (CSF) whose levels of β-amyloid _1–42_, total TAU and phospho-TAU-181 currently represent the core biomarkers in clinical practice for AD diagnosis. However, the diagnostic use of CSF biomarkers is limited due to invasive collection by lumbar puncture with potential side effects. The urgent need of less invasive, more accessible and safe biomarkers for predicting the risk of AD have led in the last decade to develop a wide research on blood biomarkers for AD risk screening, diagnosis and progression. Recently, some CSF biomarkers relevant for AD have been studied also in blood, such as p-tau181, p-tau217 and p-tau231. ^7^^–^^9^ Plasma p-tau181 seems to be predictive and specific of AD, ^10^ but its relevance for AD diagnosis and monitoring has still to be investigated longitudinally in larger cohorts. ^11^ In turn, a recent study did not shown any significant correlations between plasma p-tau181, p-tau231 and hippocampal volume. ^9^

The etiology of AD is multifactorial, involving both genetic and environmental factors, with a prominent role of oxidative stress and inflammatory disturbances that appear long before symptoms onset. Free radicals such as reactive oxygen species (ROS) generated from physiological metabolic processes, contribute, at physiological concentrations to cell cycle regulation, phagocytosis, and enzyme activation. ^12^ However, excessive generation of ROS leads to oxidative stress causing macromolecule peroxidation, Aβ metal ion redox potential and mitochondrial dysfunction. All these processes affect the cell homeostasis, the generation of ROS and the up-regulation of Aβ and p-tau formation, ^13^ contributing to the progressive deterioration of cognitive functions. The brains of patients with AD also exhibit a sustained inflammatory response that has been found in multiple postmortem studies in AD patients. ^14^ Moreover, impairments in redox and inflammatory pathways have been observed also in peripheral blood from AD patients, ^15^^–^^17^ and have been associated with low cognitive performance ^18^ and AD progression. ^15^

The advance of “omics” tools is helping in the identification of minimally invasive AD blood biomarkers and therapeutic targets within molecular pathologic networks that include redox metabolism and inflammation, integrated with brain imaging data through novel computational and statistical tools. ^19^

The aim of this study was to examine how cognitive decline and changes of hippocampal volumes are associated with redox and inflammatory changes in peripheral blood in order to identify putative blood biomarkers whose levels correlate with progression of cognitive decline and can be easily evaluated for timely disease monitoring. Accordingly, 34 individuals with different cognitive scores were analyzed using a multiparametric approach, comprising MRI measurement of different hippocampal segments and blood mRNA expression of a large panel of inflammatory and oxidative stress related genes.

## 2. Methods

### 2.1 Study cohort

34 individuals (age 56–86 years) were randomly enrolled in this cross-sectional study from the neurology departments of three medical centers in Bucharest, Romania from June 2017 to April 2019. All participants provided written informed consent for their participation and the study was approved by the local ethic committees of the participating hospitals: Clinical Hospital Colentina, 12/11.05.2017; “Prof. Dr. Al. Obregia” Psychiatry Clinical Hospital, 3/17.05.2017; “Ana Aslan” National Institute of Gerontology and Geriatrics, 299.1101.2018. The exclusion criteria comprised: (1) acute inflammatory reactions and infections in the last 30 days prior to the study inclusion; (2) history of any type of cancer and autoimmune diseases; (3) acute episodes of morbidities during the last year, before being recruited in the present study; (4) psychiatric illness. The subjects referred to the hospitals for a routine checkup or due to episodes of memory loss. All the individuals underwent neuropsychological evaluation and brain MRI. Mini–Mental State Examination (MMSE) was corrected by age and years of education (adjMMSE) as previously reported. ^20^ The individuals were categorized based on their adjMMSE in two groups: one presenting signs of cognitive decline (CD) with adjMMSE<27 (n=14), and another reporting normal cognition (NC) with adjMMSE≥27 (n=20) ( Table 1). For a subgroup of 13 subjects neuropsychological evaluation using Montreal Cognitive Assessment (MoCA) was performed at the initial visit (T0) and after 6 months (T1).

**Table 1.** Demographic and clinical characteristics of the individuals included in the study. Differences between CD and NC individuals were evaluated using the t-test for continuous and the χ ^2^ test for categorical variables.

Group (Number)	CD (N=14)	NC (N=20)	Significance
**Mean age (years±SD)**	74.7±6.55	73.6±7.36	*p*=0.646
**Sex (%F)**	64.3%	55%	χ ^2^=0.293; *p*=0.588
**Education in years (mean±SD)**	9.79±4.49	14.20±3.42	***p=0.003***
**adjMMSE (mean±SD)**	23.59±2.17	29.3±1.08	***p<0.001***
**Coffee (% consumers)**	71.42%	80%	*p*=0.562
**Body mass index (mean±SD)**	26.37±3.48	27.22±3.87	*p*=0.517
**Exposure to toxicants (% yes)**	7.1%	15%	χ ^2^=0.490; *p*=0.484
**Childhood environment (% urban)**	35.70%	70%	***χ*** ^***2***^ ***=3.927; p=0.048***
**Adult environment (% urban)**	64.3%	90%	χ ^2^=3.331; *p*=0.068
**Familiarity for AD (% yes)**	14.3%	25%	χ ^2^=0.578; *p*=0.447
**Smokers (% of smokers)** *	15.4%	26.3%	χ ^2^=0.540; *p*=0.463
**Hypertension (% affected)**	85.7 %	45%	***χ*** ^***2***^ ***=5.781; p=0.016***
**Cardiopathy (% affected)**	71.4%	5%	***χ*** ^***2***^ ***=4.371; p=0.037***
**Diabetes (% affected)**	0%	20%	χ ^2^=3.173; *p*=0.075
**Dyslipidemia (% affected)**	42.9%	20%	χ ^2^=2.072; *p*=0.150
**Hypercholesterolemia (% affected)**	50%	20%	χ ^2^=3.387; *p*=0.066
**Hypertriglyceridemia (% affected)**	7.1%	0%	χ ^2^=1.472; *p*=0.225

* Not available for one CD and one NC individual.

### 2.2 MRI acquisition

All MRI scans were performed on 1.5 Tesla Siemens Avanto machine (syngo MR B17) following the Alzheimer's Disease Neuroimaging Initiative ADNI1 protocol (adni.loni.usc.edu/methods/documents/mri-protocols/). The scan protocol included two MP-RAGE: a three-dimensional, T1-weighted gradient echo sequence and a straight axial PD/T2-weighted turbo spin echo sequence (covered below cerebellum through top of head). The following parameters were used: (a). MP-RAGE T1weighted: TR/TE/TI 2400/3.6/1000 ms, sagittal, voxel size 1.3 × 1.3 × 1.2 mm resolution; (b) T2 weighted turbo spin echo TR/TE1/TE2 3000/12/97 ms, in plane resolution 0.9 × 0.9 mm, slice thickness 3 mm, 96 slices, transversal, oriented perpendicularly to the long axis of the hippocampus, covering the whole hippocampal head, body and tail. To ensure quality scans and scanner stability after each subject scan, a quality control scan on a phantom was acquired with an additional coronal MP-RAGE.

2.2.1 Image quality control

The image quality was assessed for accuracy of FOV angulation regarding the hippocampal axes, contrast/noise of internal structure of hippocampus and motion artifacts.

2.2.2 Image processing

An open source brain image processing software FreeSurfer 6.0 available at https://surfer.nmr.mgh.harvard.edu/was used to perform the automated volumetric analyses for the structural MRI T1-weighted data and/or T2 hippocampal images. ^21^ The FreeSurfer 6.0 pipeline recon - all was run to compute the probabilistic estimated segmented volumes of the left and right whole hippocampus and of the multi-label hippocampal substructures. The hippocampal substructures that are segmented by the software are: hippocampal tail, parasubiculum, presubiculum, subiculum, CA1, CA2 + CA3, CA4, hippocampus–amygdala transition area (HATA), granule cell layer of dentate gyrus (GC-DG), molecular layer, fimbria and hippocampal fissure (not included for computing the whole hippocampal volume).

The steps in the FreeSurfer 6.0 processing perform a motion correction, non-uniform intensity normalization for intensity inhomogeneity correction, affine transformation to Talairach image space and removal of non-brain tissues. The remaining brain image volume is intensity normalized to match the FreeSurfer atlas image intensity histogram. A non-linear warping of the atlas brain image to subject brain image is used in labeling the subcortical structures. To define image features of the anatomy of the brain structures surrounding the hippocampus, a training set of 39 manually labeled 1mm T1-weighted MR scans in combination with 15 *ex vivo* scans from which hippocampal substructures were manually labeled were used to build the probabilistic atlas of hippocampal anatomy. ^21^ The atlas is represented as a tetrahedral mesh, in which each node has a corresponding vector of probabilities for the different structures encoded in the atlas.

The final segmentation output (aseg.mgz) was then used to generate a tissue classification map using the FreeSurfer Look Up Table of the segmented regions. Labels can be displayed in FreeSurfer's Freeview to assess label accuracy.

### 2.3 Gene expression analysis in blood

Venous blood (2.5 mL) was collected in PAXgene Blood RNA Tubes (Qiagen) and RNA was isolated with PAXgene blood RNA kit (Qiagen) according to the manufacturer’s protocol. RNA quantification and quality control was performed using Nanodrop 2000 spectrophotometer (Thermo Scientific). RNA (400 ng) was reverse transcribed with the RT2 First Strand Kit (Qiagen). The expression of 84 key genes involved in redox responses and of 84 genes related to inflammatory processes was evaluated with RT2 Profiler™ PCR Array Human Oxidative Stress Plus (PAHS-065Y, Qiagen, *Extended data*, Table S1 A) ^41^ and RT ^2^ Profiler™ PCR Array Human NF-κB Signalling Pathway (PAHS-025Z, Qiagen, *Extended data*, Table S1 B). ^41^ The SYBR Green chemistry on an ABI-7500 fast instrument (Thermo Fisher Scientific) was applied. The expression level of each transcript was normalized against the geometric mean of two housekeeping genes ( *HPRT1* and *RPLP0*) whose stability in blood was previously reported. ^22^ Gene expression levels were calculated as 2 ^−ΔCT^ values.

### 2.4 Statistical analysis

Statistical analysis was performed using the Statistical Package for Social Sciences, Version 17.0 (SPSS Inc) and GraphPad Prism 8. Possible demographic and clinical differences between CD and NC individuals were evaluated through the t-test for continuous and the χ ^2^ test for categorical variables. Due to the small sample size, the volumetric difference between CD and NC was evaluated by the Mann-Whitney nonparametric test. Pearson's correlation analysis was performed to correlate neuroimaging data with adjMMSE, as well as with mRNA levels in blood. All the original data used in the analysis are reported in *Underlying data.* ^41^

## 3. Results

The volume of hippocampal subfields was analyzed in connection with the registered cognitive score (adjMMSE) in a group of 34 individuals. This cohort was divided in two groups according to adjMMSE: the CD group (n=14) presenting cognitive decline (adjMMSE 17.91-26.08) and the NC group (n=20) reporting normal cognition (MMSE 27-30). The two CD and NC groups were homogenous for age, sex, and body mass index, as shown in Table 1. In terms of comorbidities, the differences between the two groups were not significant for diabetes, dyslipidemia, hypercholesterolemia and hypertriglyceridemia, but were significant for hypertension and cardiopathy. Therefore, we performed partial correlations (controlling for the effect of hypertension and/or cardiopathy) between the selected hippocampal subfields volume and adjMMSE ( *Extended data*, Table S2 A), ^41^ as well as between blood Sulfiredoxin-1 ( *SRXN1*) mRNA levels and selected hippocampal subfields volume and adjMMSE in CD individuals ( *Extended data*, Table S2 B). ^41^ The results obtained with hypertension, cardiopathy, and both hypertension and cardiopathy as control variables, were very similar to those obtained previously using bivariate correlations. This suggests that these comorbidities do not influence the found correlations between gene expression levels and selected hippocampal subfields volume and adjMMSE. Moreover, in a paper recently published by our team, aiming to identify gene expression changes that might underlie pathologic processes in elderly patients with hypertension and cardiovascular disease, the expression levels of the genes of interest were not found to be affected by these pathologies. ^22^

We found that the CD subjects presented a significant volume reduction of multiple hippocampal segments compared to NC individuals: left hippocampus, subiculum, pre-subiculum, CA1, molecular layer HP, GC ML DG, HATA and left/right CA4 ( Table 2).

**Table 2.** Significant differences of hippocampal subfields volume between the CD and NC groups. Comparisons were made using Mann-Whitney nonparametric test.

Hippocampal subfields	Group	Volume (mm ^3^)	SD	*P*-value
**Whole hippocampus left**	CD	2578.98	486.71	***0.015***
	NC	2997.21	355.77	
**Subiculum left**	CD	328.48	68.39	***0.010***
	NC	384.63	44.00	
**Presubiculum left**	CD	227.99	46.96	***0.027***
	NC	272.20	49.39	
**CA1 left**	CD	493.37	84.29	***0.017***
	NC	572.47	81.66	
**Molecular layer HP left**	CD	421.64	83.89	***0.012***
	NC	494.47	62.62	
**GC ML DG left**	CD	214.89	48.16	***0.011***
	NC	254.30	32.74	
**CA4 left**	CD	188.99	40.12	***0.023***
	NC	222.99	27.59	
**CA4 right**	CD	208.85	36.93	***0.047***
	NC	231.14	26.14	
**HATA left**	CD	41.67	11.68	***0.023***
	NC	51.00	9.32	

**Figure 1.**
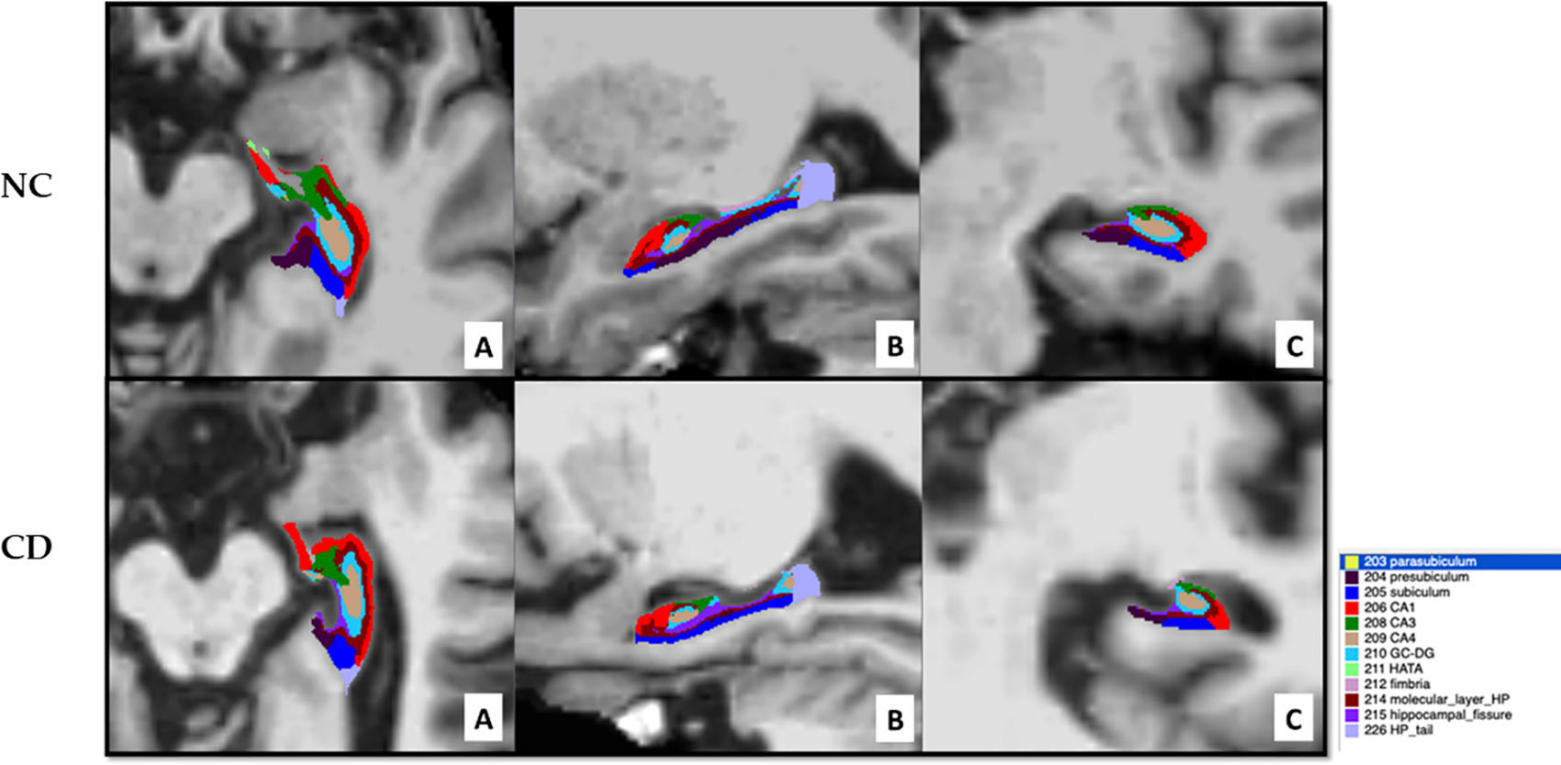
MRI scans of the hippocampus segmentation for a representative NC individual with an MMSE value of 30 (upper panel) and a representative CD patient with an adjMMSE value of 17.9 (lower panel): axial (A), sagittal (B) and coronal (C) view.

A representative axial (A), sagittal (B) and coronal (C) view of hippocampal segmentation is presented in Figure 1 for one NC individual and one CD patient, showing marked hippocampal atrophy in the CD patient.

We also correlated the volume of the selected hippocampal segments ( Table 2) with the adjMMSE values. A statistically significant positive correlation was found exclusively in the CD group ( Table 3) showing a linear association between cognitive decline and hippocampal atrophy.

**Table 3.** Correlations between selected hippocampal subfields volume and adjMMSE in all individuals, CD and NC groups. L=left side; R=right side. Correlations were established using Pearson's correlation analysis.

Hippocampal subfields		All individuals (N=34)		CD (N=14)		NC (N=20)	
		Pearson r	*P-value*	Pearson r	*P-value*	Pearson r	*P-value*
**Whole Hippocampus**	**L**	0.583	***<0.001***	0.763	***0.001***	-0.187	0.431
**Subiculum**	**L**	0.631	***<0.001***	0.765	***0.001***	0.046	0.846
**CA1**	**L**	0.486	***0.004***	0.714	***0.004***	-0.403	0.078
**Presubiculum**	**L**	0.572	***<0.001***	0.731	***0.003***	0.189	0.426
**Molecular layer HP**	**L**	0.575	***<0.001***	0.741	***0.002***	-0.187	0.430
**GC ML DG**	**L**	0.551	***0.001***	0.687	***0.007***	-0.279	0.234
**CA4**	**L**	0.554	***0.001***	0.691	***0.006***	-0.318	0.172
	**R**	0.499	***0.003***	0.793	***0.001***	-0.238	0.313
**HATA**	**L**	0.462	***0.006***	0.497	***0.071***	-0.232	0.324

The observed volume reduction was not age-dependent, considering that no significant correlations were found between the volume of the selected hippocampal segments and age ( *P*>0.05).

**Figure 2.**
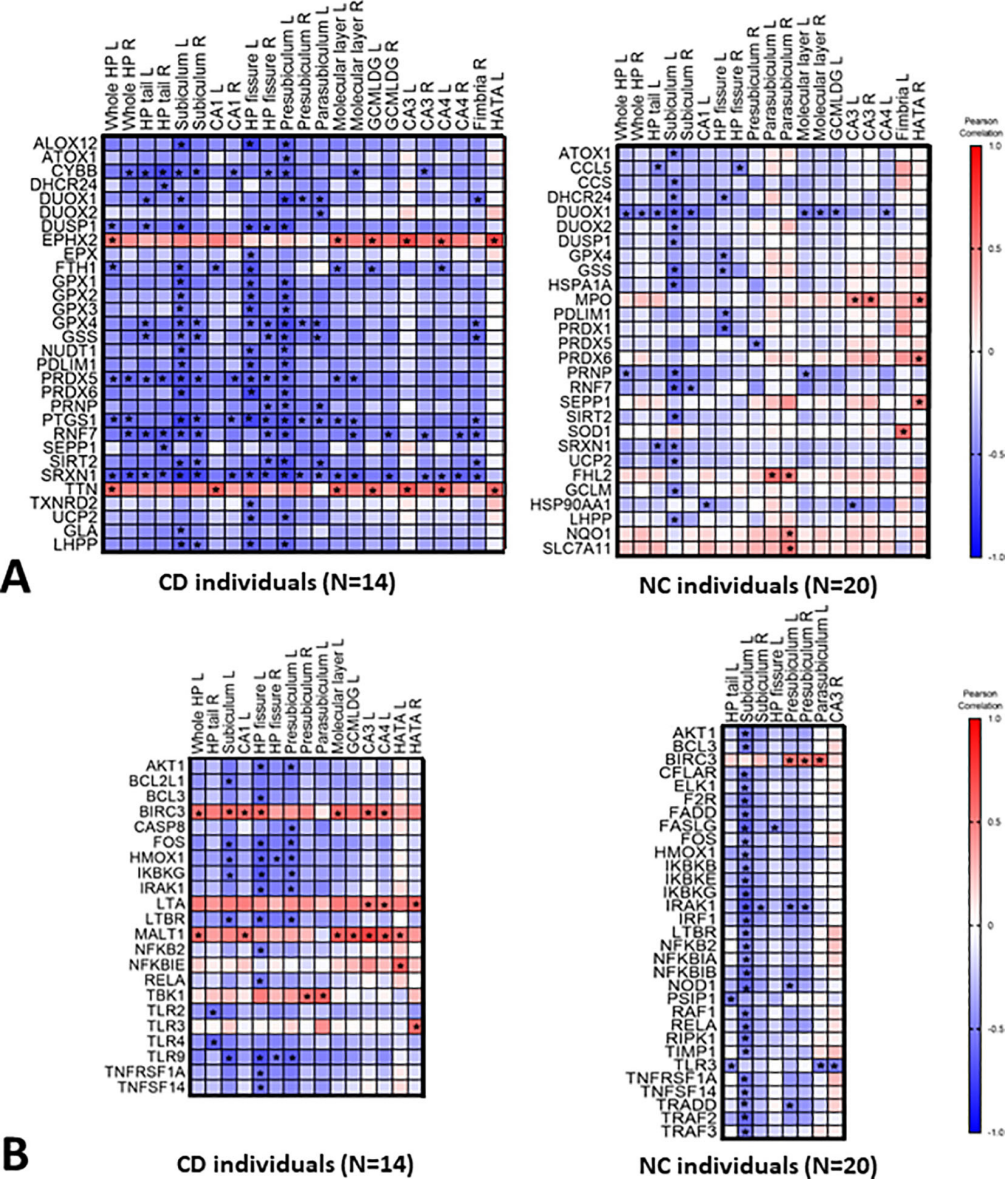
Correlation heat maps between (A) the redox genes blood levels and hippocampal segments volume; (B) the inflammatory genes blood levels and hippocampal segments volume. On the right panel are reported correlations in CD individuals and on the left in NC individuals. Stars indicate significant correlations ( *P*<0.05). Correlations were established using Pearson's correlation analysis.

**Figure 3.**
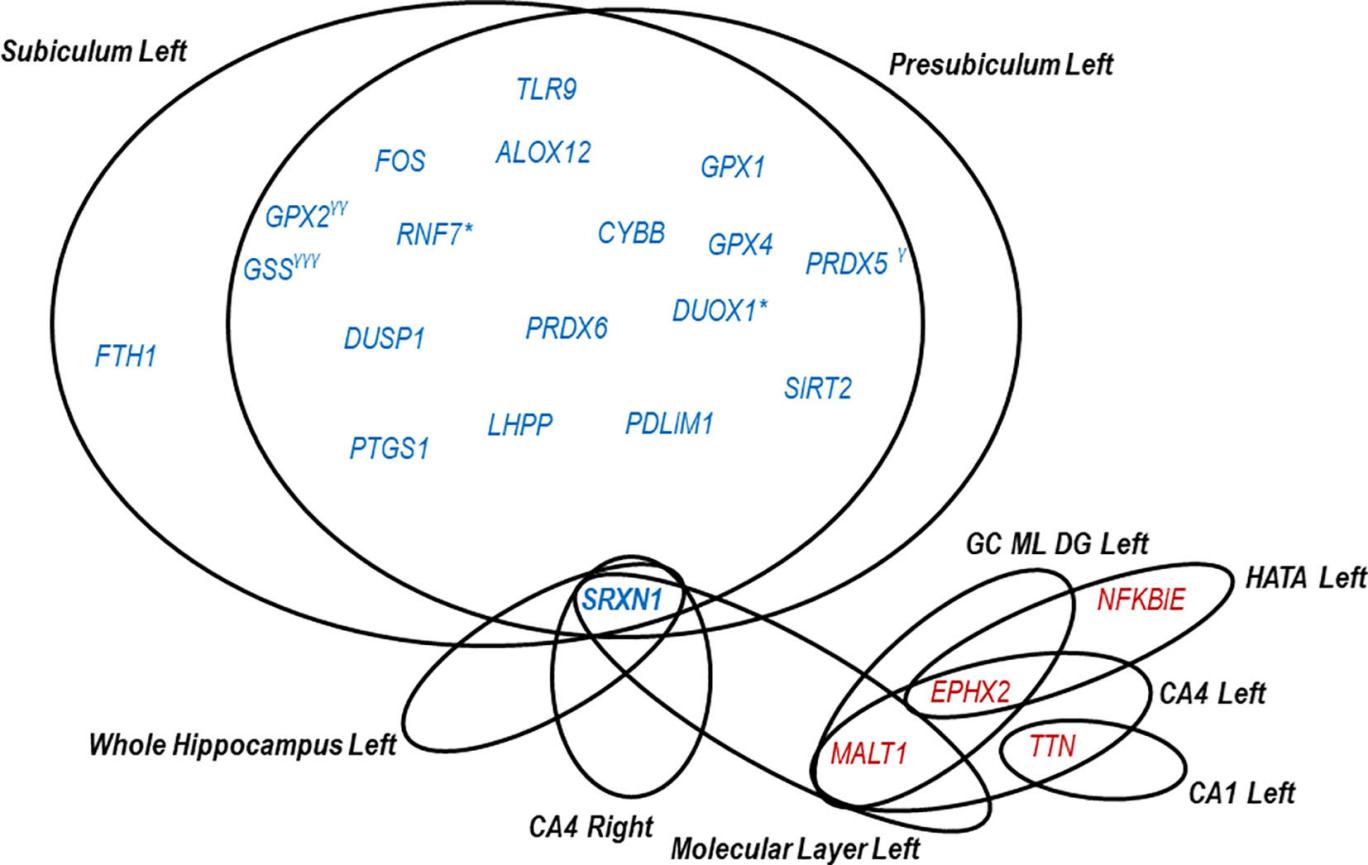
Redox and inflammatory genes significantly correlating with hippocampal segments in CD. Blue font indicates negative correlations (r<-0.60) and red font positive correlations (r>0.60). *The gene levels correlate (r<-0.60) in subiculum left both in CD and NC. γCorrelates in presubiculum left with r=-0.59. γγCorrelates in subiculum left with r=-0.55. γγγCorrelates in subiculum left with r=-0.54. Correlations were established using Pearson's correlation analysis.

We further correlated the blood expression levels of the 84 redox and the 84 inflammation genes with the volume of the selected hippocampal segments, reported in Table 2. All the correlations are presented in *Extended data*, Table S3 A-B-C. ^41^ The statistically significant correlations ( *P*<0.05) are reported in the correlation matrix shown in Figure 2A and B, and the most significant ( *P*<0.05 and -0.60 >r> 0.60) are presented in Figure 3. More specifically, 23 genes correlated with hippocampal subfields volume in the CD group ( *P*<0.05, -0.60 >r> 0.60). Most of these genes (19 out of 23) negatively correlated with left subiculum and presubiculum, suggesting that high transcript levels in blood correspond to a potentially pathologic atrophy of these brain areas. Most of the identified genes belong to redox pathways. These include oxidative stress responsive genes ( *RNF7*, *SIRT2*, *DUOX1*, *DUSP1* and *LHPP*), genes involved in ROS production ( *DUOX1*, *CYBB*), glutathione metabolism ( *GPX1*, *GPX2*, *GPX4* and *GSS*), arachidonic acid metabolism ( *GPX1*, *GPX2*, *ALOX12* and *PTGS1*), ferroptosis ( *GPX4*, *GSS*, *FTH1* and *CYBB*) and other antioxidant mechanisms ( *PRDX5* and *PRDX6*). Only four inflammatory genes ( *FOS*, *TLR9*, *ALOX12* and *PTGS1*) correlated negatively with the volume of left subiculum and pre-subiculum. Of note, *ALOX12* and *PTGS1* exhibit overlapping functions both in inflammation and in redox-mediated processes.

As shown in Figure 3, eighteen genes exhibited negative correlations with both left subiculum and presubiculum. The only gene specifically correlating with left subiculum atrophy was *FTH1.* Meanwhile, three genes strongly correlated negatively either with the left subiculum ( *PRDX5*) or presubiculum ( *GPX2* and *GSS*), without being exclusively associated with a particular hippocampal area.

While negative correlations between gene expression and volume of the left subiculum and/or presubiculum were highlighted, positive correlations were observed instead in other hippocampal segments, indicating that the atrophy of these segments is associated with a decrease in blood gene expression. Thus, two redox genes showed positive correlations with various left segments: *EPHX* correlated with left GC ML DG, CA4 and HATA, while *TTN* with left CA1 and CA4. Regarding inflammatory genes, only *NFKBIE* had a positive correlation with HATA left volume, while *MALT1* positively correlated with several left segments, including CA4, GC ML DG and molecular layer ( Figure 3).

**Figure 4.**
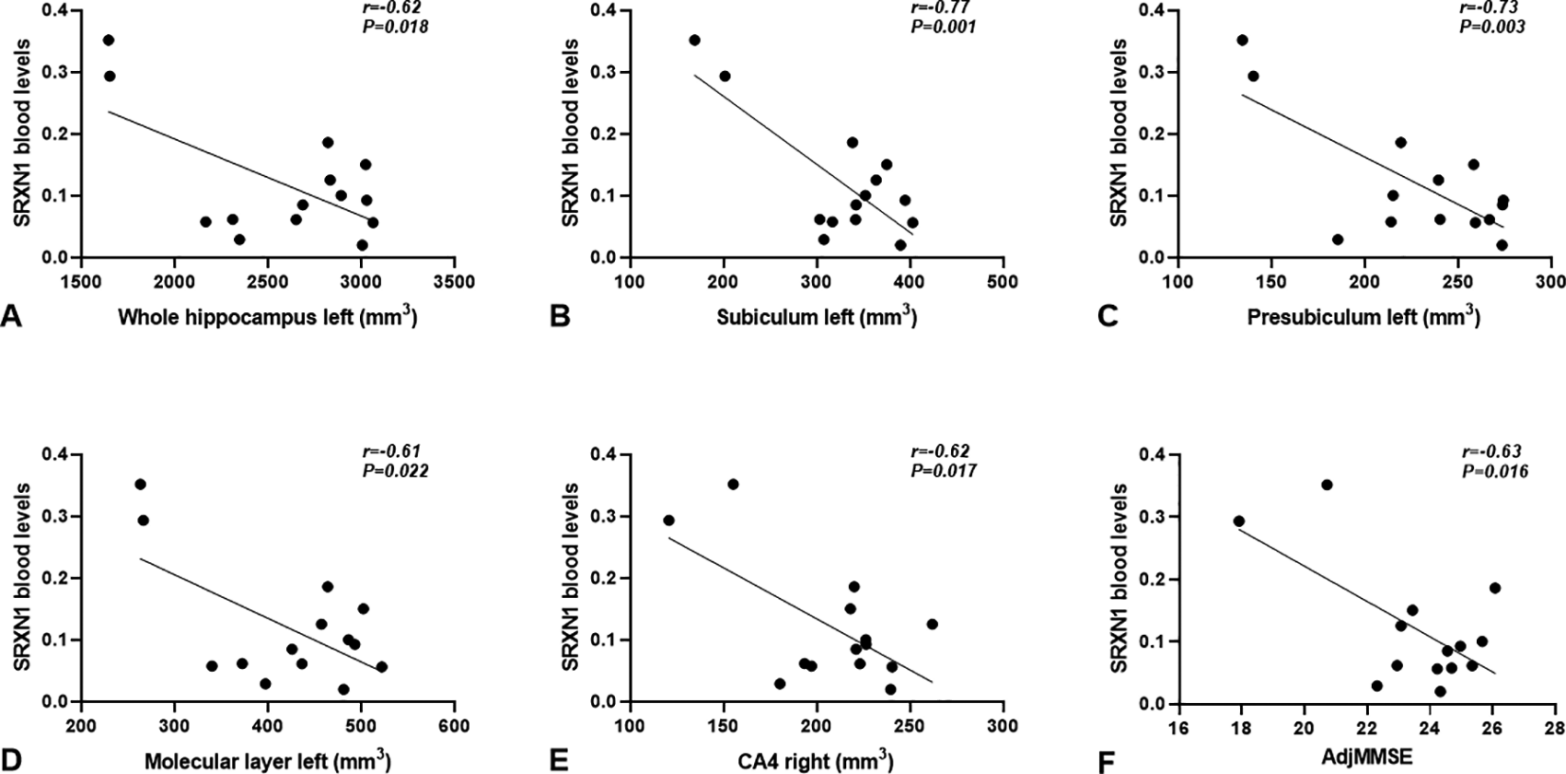
Correlations between blood *SRXN1* mRNA levels and hippocampal subfields volume, (A) whole hippocampus left; (B) subiculum left; (C) presubiculum left; (D) molecular layer left: (E) CA4 right, and (F) adjMMSE in CD individuals. *SRXN1* levels are expressed as 2 ^-∆CT^ values.

Among the genes presented in Figure 3, we emphasize the redox gene *SRXN1*, since its blood levels in CD individuals negatively correlated with the volume of many hippocampal segments. These include the left side of whole hippocampus (r=-0.62, *P*=0.018) ( Figure 4A), subiculum (r=-0.77, *P*=0.001), presubiculum (r=-0.73, *P*=0.003) and molecular layer (r=-0.61, *P*=0.022) ( Figure 4B, C, D), as well as with the right side of CA4 (r=-0.62, *P*=0.017) ( Figure 4E).

The selected genes associated with the reduction of hippocampal subfields volume ( Figure 2) were further correlated with the adjMMSE values. Only *SRXN1* significantly correlated with the adjMMSE value specifically in CD individuals (r=-0.628, *P*=0.016) ( Figure 4F), but not in NC individuals. Summarizing, our results regarding *SRXN1* pointed out that the increase of its transcript in the blood of individuals with CD is accompanied by atrophy in different hippocampal subfields, and this increase follows the cognitive decline measured with the MMSE test.

Moreover, for a subgroup of 13 individuals neuropsychological evaluation using MoCA was also performed at the initial visit (T0) and after 6 months (T1). From this subgroup six individuals presented progressive cognitive decline (MoCA mean value T0 = 20.33±4.50 and T1 = 15.91±5.46; Wilcoxon Signed Rank test, *P*=0.027), and seven remained cognitively stable. We also performed a Mann-Whitney U test between the *SRXN1* blood expression levels comparing the stable and the progressive cognitive decline groups, and although was not significant, probably due to the small number of subjects, a difference in gene expression levels was observed between the two groups in term of fold change ( *P*=0.101, FC=2.32, *Extended data*, Figure S4 ^41^). This may suggest that elevated *SRXN1* blood expression levels may precede cognitive decline.

## 4. Discussion

In this study we investigated the correlation between various hippocampal subfields volume and the expression profile of 84 redox and 84 inflammatory genes in the blood of CD and NC individuals, in the context of cognitive decline and age. We found a significant reduction in the volume of the left part of whole hippocampus and seven other segments in CD individuals compared to NC, which also correlated well with adjMMSE score, specifically in the CD group. It is worth noticing that MMSE is the most frequently used and recommended cognition test, followed by the Montreal Cognitive Assessment (MoCA) and the clock-drawing test, ^23^ and its adjustment with age and educational level is recommended. ^20^ It has been reported that atrophy of the left side of hippocampus plays a critical role in episodic verbal memory, ^24^ and may predict longitudinal decline in visuospatial function. ^25^ A meta-analysis showed a consistent left-less-than-right hippocampal volume in MCI individuals compared to controls, ^26^ and several studies reported that patients with dementia presented a higher atrophy of left hippocampus compared to the contralateral part. ^24^^,^^26^^–^^28^ Moreover, asymmetry and lateralization of hippocampal subfields in AD and MCI patients have been recently reported. ^29^

Although we did not find significant gene expression differences between CD and NC, it appears that a volume decrease in hippocampal segments was well correlated with the transcript levels of 23 blood genes in the CD group. The highest number of correlations was observed with the subiculum and presubiculum left, which is considered to be the earliest hippocampal anatomical marker of AD. ^30^ Most of the correlating genes are involved in redox regulation, emphasizing that changes in the redox balance seem to be relevant in early stages of disease. Multiple lines of evidence have shown that oxidative stress is the earliest event in AD that precedes by decades the onset of clinical dementia. During the diseases progression, this mechanism is accompanying by compensatory responses such as the induction of anti-oxidant responses that may provide some protective mechanisms to ensure that neuronal cells are not irreversibly damaged by the oxidative insult. ^31^

The negative correlations between the transcript levels of several redox genes and the hippocampus segments volume suggested that in CD patients, a potential increase of the oxidative activity in the blood of CD patients correlated with hippocampal atrophy, especially in the left subiculum and presubiculum. The identified redox genes are known to be involved in ROS production ( *DUOX1*, *CYBB*) and in arachidonic acid metabolism ( *GPX1*, *GPX2*, *ALOX12* and *PTGS1*), as well as in antioxidant responses, encompassing peroxiredoxins ( *PRDX5* and *PRDX6*), genes involved in glutathione metabolism ( *GPX1*, *GPX2*, *GPX4* and *GSS*) and other redox-regulated genes ( *RNF7*, *SIRT2*, *DUOX1*, *DUSP1* and *LHPP*). Three of these genes ( *GPX2*, *FTH1* and *SRXN1*) are targets of the transcription factor Nrf2 which is the master inducer of antioxidant responses against oxidative and electrophilic challenges. ^32^ Alteration of the redox balance due to defective Nrf2 activity has been associated with cognitive impairment. ^33^ As such, hippocampal atrophy in early stages of disease seem to be associated with an antioxidant response in blood, aimed to restore redox homeostasis. In turn, *SIRT2*, a member of the sirtuin family with a pathological role in AD, negatively correlating with hippocampal volume, could be therapeutically downregulated to improve cognitive abilities in AD patients. ^34^ The negative correlation of the left subiculum and presubiculum volume with the levels of the dual specificity phosphatase *DUSP1,* ^35^ involved in the pro-inflammatory toll-like receptor signaling, ^36^ along with *FOS*, a gene involved in cell proliferation, differentiation, transformation, and apoptotic cell death, ^37^ suggests that even mild hippocampal atrophy might be associated with systemic inflammatory responses in blood.

One of the most interesting findings of this work is the strong negative correlations between *SRXN1* blood transcript levels, hippocampal subfields atrophy and cognitive impairment. To our knowledge, this is the first study that highlights *SRXN1* as a useful blood-based biomarker that is able to reflect in blood the cognitive decline accompanied by hippocampal atrophy in patients with signs of memory loss. The neuroprotective effect of SRXN1 has been shown in several *in vitro* and *in vivo* studies, and SRXN1 it has been proposed as a therapeutic target for chronic neurodegenerative diseases. ^38^^–^^40^ The *in vitro* studies performed so far on the connection between SRXN1 and CNS have shown that SRXN1 can protect nerve cells from oxidative damage induced by hydrogen peroxide due to its antioxidant and anti-apoptotic action. ^41^ Furthermore, it has been shown that SRXN1 may protect astrocytes from H _2_O _2_-induced oxidative stress injury by activating the Notch signaling pathway, ^42^ and to protect cells against mitochondrial dysfunction. ^43^ Additionally, other *in vitro* studies on spinal cord neurons demonstrated that SRXN1 mRNA and protein overexpression has a positive impact by attenuating oxidative damages and decreasing neuronal apoptosis. Moreover, it seems that the cytoprotective transcription factor Nrf2 is directly controlling the expression levels of the antioxidant *SRXN1* gene in astrocytes. ^44^ Most probably, the negative correlation between *SRXN1* transcript levels and pathologic brain atrophy derives from an enhanced systemic oxidative stress in CD patients which triggers the activation of various antioxidant mechanisms, such as those mediated by *SRXN1.*

The findings of this study indicate that volume changes in hippocampal segments are negatively correlating with the blood transcript levels of 19 genes, most of them being involved in redox regulation. Significant correlations were found mainly with the left part of subiculum and presubiculum of individuals with cognitive decline. Our results particularly highlight the *SRXN1* gene, whose mRNA blood levels exhibited a tendency to increase with cognition decline and with the level of the hippocampal atrophy in five segments. *SRXN1* might be a valuable candidate blood biomarker for non-invasively monitoring in the blood the evolution of hippocampal atrophy in patients with mild cognitive decline. The limitations of this study are the A major limit of this study is the small number of the investigated cases and the evaluation of only 84 key genes involved in redox responses and of 84 genes related to inflammatory processes, using target arrays. Results should be further validated in a larger and longitudinal cohort.

## Data availability

### Underlying data

Harvard Dataverse: Underlying data for ‘SRXN1 blood levels negatively correlate with hippocampal atrophy and cognitive decline’. https://doi.org/10.7910/DVN/I2UBIF . ^45^

### Extended data

Harvard Dataverse: Extended data for ‘SRXN1 blood levels negatively correlate with hippocampal atrophy and cognitive decline’. https://doi.org/10.7910/DVN/I2UBIF . ^45^

Data are available under the terms of the Creative Commons Zero “No rights reserved” data waiver (CC0 1.0 Public domain dedication).

## Author contributions

CAC and EM designed and coordinated the study. MD, CAC, IC and GN performed the laboratory experiments, contributed to data processing and writing. BOP, CT and LS were responsible of the collection and diagnosis of the samples included in the study. EM and CAC were responsible of data processing, statistical analysis and wrote the first draft of the manuscript. GM gave her contribution in critical revision, data interpretation and writing. All authors discussed the results, commented on the manuscript and agreed to the published version of the manuscript.
