# Supplementary material for: Sulfiredoxin-1 blood mRNA expression levels negatively correlate with hippocampal atrophy and cognitive decline
Source: F1000Res. 2022 Mar 21;11:114. Originally published 2022 Jan 28. [Version 2] doi: 10.12688/f1000research.76191.2 (PMC8857523; doi:10.12688/f1000research.76191.2)
Supplement: Peer review history

# Peer review: Reviewer response for version 1

**DOI:** 10.5256/f1000research.80158.r121476

**Published:** 2022-02-18

**Authors:** Sermin Genc

**Affiliations:** 1 International Biomedicine and Genome Center, İzmir, Turkey

In this manuscript, Milanesi *et al.* analyzed the relationship between cognitive decline and hippocampal subfield atrophy and redox and inflammatory gene expression in the blood. The study is well organized and the results are presented quite clearly. I disagree with the first reviewer's opinion on the necessity of RNA sequencing. I think that targeted analyzes are more informative than large scale analyses.

 Although the number of cases included in the study was small, the inclusion and exclusion criteria were clearly defined. In addition, I found the corrections of MMSE scores according to age and education very important for the reliability of the results.

 The authors performed volumetric analyses of hippocampal substructures by using MRI. While measuring the total hippocampal volume in general, the evaluation of hippocampal substructures is one of the strengths of the study.

 Finally, blood expression levels of several genes (redox and inflammation) were evaluated by qPCR.  Inflammation and oxidative stress are two important mechanisms involved in the pathogenesis of neurodegenerative diseases such as Alzheimer's with cognitive dysfunction. For this reason, I considered it a correct approach to perform gene expression analysis targeting these two mechanisms.

 An important issue here is how peripheral gene expression changes occur in a situation affecting the central nervous system. Perhaps this issue should be considered in the discussion.

Is the work clearly and accurately presented and does it cite the current literature?

Yes

If applicable, is the statistical analysis and its interpretation appropriate?

Yes

Are all the source data underlying the results available to ensure full reproducibility?

Yes

Is the study design appropriate and is the work technically sound?

Yes

Are the conclusions drawn adequately supported by the results?

Yes

Are sufficient details of methods and analysis provided to allow replication by others?

Yes

Reviewer Expertise:

Neuroscience, Neurobiology, Alzheimer's disease, biomarker

I confirm that I have read this submission and believe that I have an appropriate level of expertise to confirm that it is of an acceptable scientific standard.

# Peer review: Reviewer response for version 1

**DOI:** 10.5256/f1000research.80158.r121474

**Published:** 2022-01-31

**Authors:** David Gurwitz

**Affiliations:** 1 Department of Human Molecular Genetics and Biochemistry, Sackler Faculty of Medicine, Tel Aviv University, Tel-Aviv, Israel

This is a well-perceived and accomplished study reporting on correlations between cognition status (MMSE scores), hippocampal volumes (MRI), and blood mRNA expression levels of redox and inflammatory genes (Qiagen PCR Array Human Oxidative Stress Plus). The conclusions are convincingly supported by the reported findings, which (albeit requiring validation by larger cohorts) add to our understanding on the biology of dementia - in particular regarding oxidative stress and inflammation. 

 The article would benefit from addressing the following minor points.

1. The title should be edited to reflect the fact that mRNA levels were measured (rather than just " *SRXN1* blood levels "). Additionally, I think it would be better to use the full gene name, Sulfiredoxin-1, rather than its abbreviation, in the title. I, therefore, suggest changing the title to " Sulfiredoxin-1 blood mRNA expression levels negatively correlate with hippocampal atrophy and cognitive decline".
2. The full *SRXN1* gene name, Sulfiredoxin-1, should be used (followed by its gene symbol) also in the Abstract and for the first time that it is mentioned in the main text.
3. The authors conclude in the last sentence of their Discussion that a "major limit of this study is the small number of the investigated cases. Results should be further validated in a larger and longitudinal cohort." They should also mention that the use of commercial microarrays for only " 84 key genes involved in redox responses and of 84 genes related to inflammatory processes". Future studies should ideally attempt to validate the present findings not only with larger cohorts but also with RNA-seq.
4. The discussion on the relevance of *SRXN1* to dementia should be expanded by citing at least some of the following published studies:

- Wu J *et al* 2017. Neuroprotective effects of sulfiredoxin-1 during cerebral ischemia/reperfusion oxidative stress injury in rats. ^1^
- Yu *et al* 2015. Sulfiredoxin-1 protects primary cultured astrocytes from ischemia-induced damage. ^2^
- Sunico *et al* 2016. Role of sulfiredoxin as a peroxiredoxin-2 denitrosylase in human iPSC-derived dopaminergic neurons. ^3^
- Wu *et al* 2012. c-Jun-dependent sulfiredoxin induction mediates BDNF protection against mitochondrial inhibition in rat cortical neurons. ^4^

Is the work clearly and accurately presented and does it cite the current literature?

Partly

If applicable, is the statistical analysis and its interpretation appropriate?

Yes

Are all the source data underlying the results available to ensure full reproducibility?

Yes

Is the study design appropriate and is the work technically sound?

Yes

Are the conclusions drawn adequately supported by the results?

Yes

Are sufficient details of methods and analysis provided to allow replication by others?

Yes

Reviewer Expertise:

RNA biomarkers for neuropsychiatric disorders

I confirm that I have read this submission and believe that I have an appropriate level of expertise to confirm that it is of an acceptable scientific standard.

## References

[1] : Neuroprotective effects of sulfiredoxin-1 during cerebral ischemia/reperfusion oxidative stress injury in rats. *Brain Res Bull* .132: 10.1016/j.brainresbull.2017.05.012 99-108 10.1016/j.brainresbull.2017.05.012 28552673

[2] : Sulfiredoxin-1 protects primary cultured astrocytes from ischemia-induced damage. *Neurochem Int* .2015;82: 10.1016/j.neuint.2015.01.005 19-27 10.1016/j.neuint.2015.01.005 25620665

[3] : Role of sulfiredoxin as a peroxiredoxin-2 denitrosylase in human iPSC-derived dopaminergic neurons. *Proceedings of the National Academy of Sciences* .2016;113(47) : 10.1073/pnas.1608784113 E7564-E7571 10.1073/pnas.1608784113 PMC512729927821734

[4] : c-Jun-dependent sulfiredoxin induction mediates BDNF protection against mitochondrial inhibition in rat cortical neurons. *Neurobiol Dis* .2012;46(2) : 10.1016/j.nbd.2012.02.010 450-62 10.1016/j.nbd.2012.02.010 22402332
